# Novel High-Efficiency Single-Site Rare Earth (RE) Catalyst System for Isoprene Polymerization

**DOI:** 10.3390/polym17091219

**Published:** 2025-04-29

**Authors:** Di Kang, Rongqing Ma, Hongfan Hu, Yi Zhou, Guoliang Mao, Shixuan Xin

**Affiliations:** 1 Provincial Key Laboratory of Polyolefin New Materials, College of Chemical Engineering, Northeast Petroleum University, Daqing 163318, China; kangdi1992@outlook.com (D.K.); maoguliang@nepu.edu.cn (G.M.); 2 PetroChina Shanghai Advanced Materials Research Institute Co., Ltd., 1 Shengang Avenue, Lingang District, Shanghai 201306, China; mrq@petrochina.com.cn; 3 PetroChina Petrochemical Research Institute, 7 Kunlun Road, Changping District, Beijing 102206, China; huhongfan@petrochina.com.cn (H.H.); zhouyi9@petrochina.com.cn (Y.Z.)

**Keywords:** tridentate ligand, rare-earth metal, polyisoprene, rubber

## Abstract

Bis-(*o*-dipheylphosphinophenyl)amine, a tridentate (PNP) chelating ligand, and several of their Rare Earth (RE) metal complexes, [bis-(*o*-dipheylphosphinophenyl)amido]-RER_2_, {[(C_6_H_5_)_2_P-*o*-(C_6_H_4_)]_2_NMR_2_ (R = -CH_2_-*o*-(C_6_H_4_)NMe_2_: M = Y, **1**; Nd, **2**; Gd, **3**;), are prepared in high yields. When activated with the strong Lewis acid MMAO-7, all these complexes exhibit catalytic activity toward the polymerization of isoprene (IP) in non-protic hydrocarbons. While the Nd complex (**2**) showed moderate activity and stereoselectivity, the Y and Gd complexes (**1** and **3**) exhibited extremely high catalytic efficiency in IP homo-polymerization, and produced polyisoprene rubber (PI) with 95% to over 99% *cis*-1,4 stereoselectivity and narrow polydispersity indices (<2.0). Moreover, under industrially relevant conditions, complex **3** can catalyze IP to produce ultrahigh molecular weight PI (UHMW-PI, MW up to 1200–2600 kg/mol) rubber with a very narrow polydispersity index (PDI ca. 1.1–1.6), a high-performance elastomeric material mimic of natural rubber (NR).

## 1. Introduction

The transition metal-mediated polymerization of 1,3-butadiene and isoprene is one of the well-developed industrial processes, as the elastomeric polymers (polybutadiene, PB, and polyisoprene, PI) possess physical and mechanical properties that can facilitate practical applications in truck and car tires, footwear, hoses, belts, cables, modification of synthetic resins, and construction. RE metal coordination polymerization catalytic systems can provide conjugated diene rubbers with controllable microstructure, manageable molecular weight and narrow PDI (ca. 1–3), and high productivity, which exhibit desirable polymer physical and mechanical properties and eco-friendly production processes [1].

By modification of the substituents of the organic moieties on transition metal complexes, a single-site catalyst (SSC)-mediated coordination polymerization system can control the microstructure of the resulting polymeric materials in a rational way, thus providing a useful tool for the production of high-performance polymer materials that cater to modern society’s ever-growing demand for new materials [2]. The RE metal SSC-mediated regio- and chemo-selective polymerization of 1,3-conjugated dienes had a significant effect on the elastomeric polymers’ stereoregularity, molecular weight, molecular weight distribution, and chain end groups, which are pivotal elements in determining the physical and mechanical properties and end use of the materials [3,4,5].

The study of RE metal SSC polymerization of isoprene (IP) provides a wide range of PIs that possess physical and mechanical properties which are poles apart, and the highly *cis*-1,4-PI is a material that has outstanding flexibility and ductility similar to vulcanized natural rubber, which affords easier processing and recycling, and is widely applied in the manufacturing of gloves, hoses, conveyor belts, adhesives, sports equipment, tires, etc. [6,7,8,9]. Therefore, precise control of the microstructure of PIs with specific properties is an appealing and timeless subject [10].

PIs are generally classified into *cis*-1,4-PI, *trans*-1,4-PI, 3,4-PI, and their possible microstructural combinations, depending on the catalyst systems and processes that specified to the regio-selective and stereoselective polymerization of IP [8]. Among them, high *cis*-1,4-PI has a near identical microstructure and main chain topology to natural rubber’s (NR) predominant component. Thus, *cis*-1,4-PI is one of the well-studied conjugated diene rubbers, and its molecular weight, PDI, and particularly *cis*-1,4-regularity and other microstructures have important effects on their rheological and mechanical properties [11,12].

Due to the limited supply of NR and the increased demand for high-performance synthetic conjugated diene rubbers [13,14,15,16], developing coordination polymerization catalytic systems and processes that possess catalytic properties with practically high catalytic productivity, high stereoselectivity, high molecular weight, and a narrow PDI is the most prioritized demand and effort. Although significant advances have been achieved in RE metal catalyst systems for the polymerization of 1,3-conjugated dienes [12,17], the development of practical industrial production of high *cis*-1,4-PIs that mimic NR is still in prioritized demand [18].

In recent years, several research groups developed RE metal coordination polymerization catalyst systems that showed high potentials for practical industrial application. For instance, the [(PNPPh)Y(CH_2_SiMe_3_)_2_(THF)/[PhMe_2_NH][B(C_6_F_5_)_4_] catalytic system exhibited high activity with *cis*-1,4 selectivity up to 99.6% [19]; the [(S,S)-Phebox-^i^Pr]YCl_2_(THF)_2_/[PhNHMe_2_][B(C_6_F_5_)_4_]/Al^i^Bu_3_ ternary system demonstrated high activity and outstanding *cis*-1,4-selectivity (up to 99.5%) [4]; the (1,3-bis(oxazolinymethylidene)isoindol)Y(CH_2_SiMe_3_)_2_/[Ph_3_C][B(C_6_F_5_)_4_]/Al^i^Bu_3_ ternary system showed high *cis*-1,4 selectivity (up to 97%) [20]; the yttrium complexes bearing [PNNox] ligands/ [Ph_3_C][B(C_6_F_5_)_4_] had high catalytic activities and afforded PI with 96.2–99.0% *cis*-1,4 units [21]. Several other RE metal SSC catalyst systems for IP polymerization are also reported [13,15,16,20,22,23,24,25,26,27].

In essence, the abovementioned RE metal SSC systems are operated in relatively polar solvents such as chlorobenzene (dielectric constant 5.62 F/m) or aromatic solvents such as toluene (dielectric constant 2.40 F/m). A few known reports exhibited relatively poor catalytic activity and selectivity in the aliphatic solvents, mainly due to the poor solubility of the cationic RE^3+^ metal species in saturated hydrocarbons (a commonly used hydrocarbon solvent in academic research, n-hexane, has a dielectric constant of 1.87 F/m). Thus, it is practically important to develop catalyst systems that can easily be adopted in current industrial processes, i.e., they should have low cost, high catalytic activity, high stereoselectivity, and good thermal stability in aliphatic media to meet industrial production conditions [28].

Currently, much attention had been paid to improving the catalyst system by tuning the stereoelectronic properties of the auxiliary ligands [29,30]; however, the intrinsic poor solubility of [PhNHMe_2_][B(C_6_F_5_)_4_] and the RE^3+^ metal cations in saturated hydrocarbons is the major obstacle for these borate-activated RE^3+^ metal SSC systems to be practically adoptable in current industrial process. On the other hand, the MAO-activated metallocene cationic systems have been widely used in industrial processes for the production of high-valued polyolefins, and the PNPRE/MMAO-3A/Al^i^Bu_3_ (RE = Y, Nd, Gd) catalytic system has demonstrated high catalytic activity toward IP in hexane solvent, with *cis*-1,4-selectivities ranging from 91–95% [31].

We report now an aliphatic hydrocarbon soluble and thermally stable RE^3+^ metal SSC catalytic system which consists of a ternary PNPRE/MMAO-7/Al^i^Bu_3_ RE^3+^ metal catalyst system, {PNP = [bis-(*o*-dipheylphosphinophenyl)amido], [(C_6_H_5_)_2_P-*o*-(C_6_H_4_)]_2_N}MR_2_ (R = -CH_2_-*o*-(C_6_H_4_)NMe_2_: M = Y, **1**; Nd, **2**; Gd, **3**;)}, for isoprene polymerization under industrially relevant conditions. The catalytic system showed very high catalytic activity, high *cis*-14 stereoselectivity (96% to over 99%) for the polymerization of *cis*-1,4-PI, and most significantly, the system can produce ultrahigh molecular weight PI (UHMW-PI, 1.0–2.6 × 10^6^ g/mol) with over 99% *cis*-1,4 content, a mimic material of natural rubber.

## 2. Materials and Methods

All synthetic procedures for the preparation of RE^3+^ metal complexes and the experiments for polymerization of isoprene were conducted under standard Schlenk technique conditions. The vacuum oven-dried (120 °C) Schlenk-type glassware was connected with a Schlenk line (10^−5^ Torr); the vacuum/high-purity nitrogen (O_2_ and H_2_O ≤ 1.0 ppm) refilling circle was repeated 3 times prior to carrying out the synthetic and polymerization procedures, or the reactions were carried out in a high-purity Ar gas atmosphere glovebox (Vigor), in which the O_2_ and H_2_O were kept below 0.1 ppm.

Isoprene was purchased from Sinopharm Chemicals or Alfa Aesar, and diluted with hexane to a 1.0 M/L solution; triisobutyl aluminum (TiBAL) 100 ppm (weight %) was added to react with the inhibitor, and it was stored in a −20 °C refrigerator inside the glovebox for at least 48 h prior to use.

^n-^Butyllithium (1.0 M solution in n-hexane), TiBAL, diethylzinc and diisobutyl aluminum hydride (HDiBAL) were purchased from Beijing Bailingwei Technology Co., Ltd. and used as received. ^n-^butylethylmagnesium (^n^BuMgEt) and BHT were purchased from Acros Organics and used as received. Chemicals and organic reagents were purchased from Sigma-Aldrich company/Sinopharm Chemicals and used as received. Bis-(o-dipheylphosphinophenyl) amine, [(C_6_H_5_)_2_P-o-(C_6_H_4_)]_2_NH, was synthesized following the literature procedures on a large scale and stored in the glovebox for sufficient ligand supply [32]. The solvents used for preparing RE metal complexes were purified by passing through a serial column packed with 4A molecular sieves and R3-11 catalyst (BASF), which kept the O_2_ and H_2_O ≤ 1.0 ppm.

MMAO-7 was purchased from Akzo Nobel and used as received. YCl_3_, NdCl_3_ and GdCl_3_ were purchased from Sigma Aldrich, and were used as received.

Nuclear magnetic resonance (NMR) spectra for ^1^H, ^13^C, ^31^P were measured on a Bruker Avance 400 Ultrashield^TM^ spectrometer (Bruker), and the chemical shifts were referenced to the deuterated solvents’ residual resonances (proton resonances: CDCl_3_, 7.26 ppm; C_6_D_6_, 7.16 ppm; CD_2_Cl_2_, 5.32 ppm; Toluene-d_8_, 2.08/6.97/7.01/7.09 ppm; THF-d_8_, 1.72/3.58 ppm; acetone-d_6_, 2.05 ppm; D_2_O, 4.79) and/or the internal standard tetramethyl silane (TMS) signal (0.00 ppm). The deuterated NMR solvents used for the RE complexes were rigorously deoxygenated and demoisturized, kept in glovebox prior to use. The solvents used for preparing rare-earth complexes (hexane, diethyl ether, toluene, THF, CH_2_Cl_2_) were purified by utilizing a 5-channel solvent purification unit, which kept the solvents O_2_ and O_2_ and H_2_O ≤ 1.0 ppm.

Elemental analysis was performed on a UNICUBE elemental analyzer (Elementar, Langenselbold, Germany).

Gel permeation chromatography (GPC) spectra were measured on an Agilent 1260 Infinity II chromatograph (Agilent Technology Co., Ltd.), equipped with RI, LALLS and viscometry (VS) detectors. The columns were calibrated with polystyrene standards by use of the universal calibration methods [33,34,35]. The sample preparation and measurement methods are as follows: All PI samples were dried in a vacuum oven under dynamic vacuum to constant weight. PI samples (5~10 Mg) were weighed out and dissolved in THF, and the solutions were passed through a 5 μm filter to remove insolubles. GPC measurements were operated at 40 °C with a THF eluent flow rate of 1.0 mL/min, and recorded with RI, LALLS and VS detectors.

The Tg of PI samples was measured on a NATZSCH DSC 3500 Sirius differential scanning calorimeter (DSC). The PI samples were cooled down to −140 °C with cooling rate of 10 °C/min, held for 5 min, and then heated at rate of 5 °C/min to 40 °C. The turning point of the glass transition temperature range was taken as the PI’s Tg.

### The Polymerization of Isoprene

A typical process for isoprene polymerization: Isoprene (1.0 M solution in n-hexane, 10 mL, 10.0 mmol) was added to a 100 mL Schlenk flask in a glove box, followed by the addition of triisobutyl aluminum (0.1 M solution in hexane, 0.5 mL, 0.05 mmol), sealed with a rubber septum, and stirred at room temperature for 30 min. Then, the RE metal complex **3** (0.01 M solution in toluene, 0.5 mL, 0.005 mmol) and MMAO-7 (10 wt% in heptane, Al/Gd ≥200 equivalent) were added in sequence into the reaction flask with gas-tight micro-liter syringes, respectively. The flask was placed in the oil bath at the specified temperature, and the mixture was stirred with a constant torque magnetic stirrer within the specified time. The reaction was terminated by addition of 0.5 mL of 2,6-di-tert-butyl-4-methylphenol (BHT) (0.1 M solution in toluene/ethanol). The reaction flask was removed from the glove box, the PI was precipitated with ethanol at room temperature, washed with deionized water for 3 times, and then with ethanol for 2 times. The sticky PI raw material was put into a glass vial and dried in a vacuum oven under dynamic vacuum to constant a weight.

## 3. Results and Discussion

### 3.1. Preparation of the Tridentate PNP-Ligated Rare-Earth Metal Complexes **1**–**3**

**Scheme 1:**
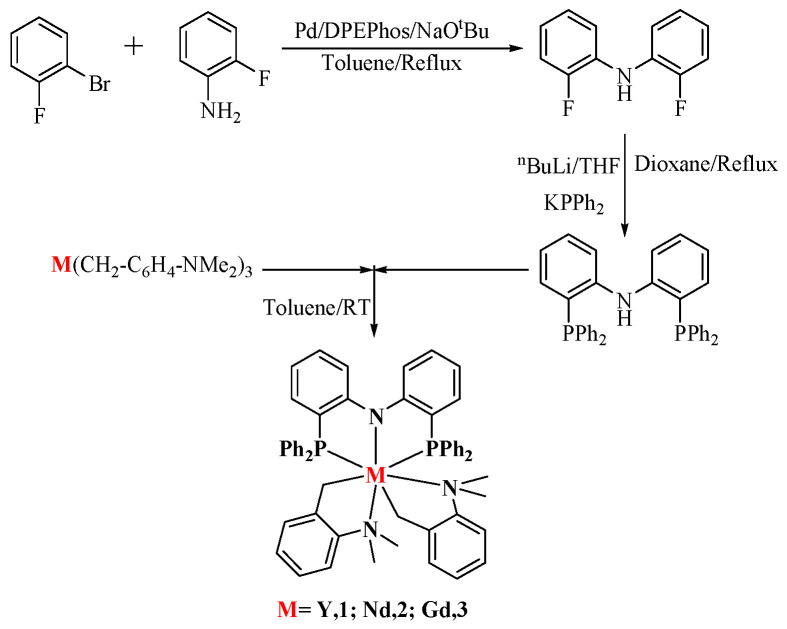
Synthesis of the PNP-ligated rare-earth metal complexes **1**–**3**.

The PNP-ligated rare earth metal complexes **1**–**3** are prepared with a modified literature method [32] and the synthetic procedures are summarized in Scheme 1. The representative proton and P-31 NMR spectra for the diamagnetic complex **1** are presented in Supporting Information (see Supporting Information Figure S1) to demonstrate the complex purity level is sufficiently high for carrying out the subsequent polymerization experiments, while the purity measurements for the paramagnetic complexes **2** and **3** are described elsewhere (see Supporting Information S1).

### 3.2. Polymerization of Isoprene with MMAO-7 Activator in Hexane and Toluene

A mixture of complexes **1**–**3** with commercially available MMAO-7 (10 wt% in heptane) and Al^i^Bu_3_ forms a soluble homogeneous catalyst system in hexane and toluene at ambient temperature. IP polymerization results with values averaged over three runs using the complexes **1**–**3**/MMAO-7/Al^i^Bu_3_ ternary catalytic system are compiled in Table 1.

Polymerization results (Table 1) showed that complex **2**/MMAO-7/Al^i^Bu_3_ exhibited moderate catalytic activity (Run 2) when compared with complexes **1** and **3**, and relatively high *cis*-1,4 stereospecific selectivity combined with a small amount of *trans*-1,4- and 3,4-units (*cis*-1,4-unit, 96.1%; *trans*-1,4-unit, 1.3% and 3,4-unit 2.6%). Under identical polymerization conditions, complexes **1** and **3** polymerized IP with very high to ultrahigh catalytic activity and could reach over 99% *cis*-1,4-selectivity in hexane in a wide temperature range. Moreover, the **3**/MMAO-7/Al^i^Bu_3_ catalytic system can initiate IP polymerization in a short period at temperatures as low as 0 °C (IP/**3** = 2000) to reach complete IP monomer conversion in less than 5 min. The polymerization reaction can be operated in a wide temperature range (0–80 °C), indicating that the catalytic system has intrinsic high thermostability.

It is interesting to notice that when the polymerization is carried out in an aromatic solvent (Run 15 in Table 1), toluene, it does not show an obvious advantage in the aspects of catalytic activity and molecular weight (compared with Run 13 under identical conditions), except for being slightly higher in stereoselectivity, suggesting that the **3**/MMAO-7/Al^i^Bu_3_ catalytic system maintained a homogeneous SSC nature in saturated hydrocarbon media, a desirable advantage for the system to be adopted into the current industrial processes.

**Figure 1:**
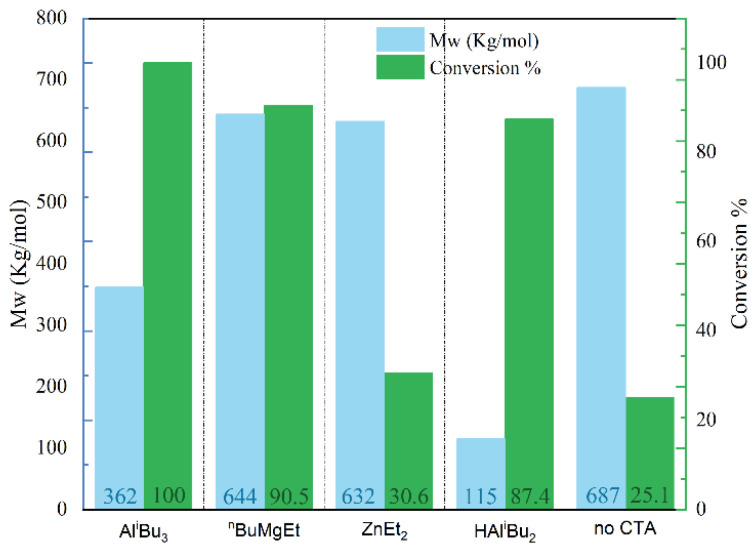
Effects of chain transfer agents on molecular weight and conversion.

When potential chain transfer agents (CTAs), such as n-butylethyl magnesium, ^n^BuMgEt (Run 4), diethylzinc, ZnEt_2_ (Run 5), diisobutyl aluminum hydride, and HAl^i^Bu_2_ (Run 6) were applied in the polymerization process, only the HAl^i^Bu_2_ showed clearly the chain transfer effect, as it enhanced the catalytic activity (with a conversion of 87.4% in Run 6 compared with a conversion of 25.1% in Run 7), and the molecular weight decreased significantly (115 kg/mol in Run 6 compared with Runs 4, 5, and 7, Mw 632~687 kg/mol), as well as a broadened PDI (1.74 in Run 6 compared with 1.19 in Run 7). It is noticeable that the Grignard reagent showed obvious enhancement in catalytic activity (conversion over 90% in Run 4 compared with 25.1% in Run 7); however, the molecular weight, PDI, and stereoselectivity remained marginally changed compared with the blank run (Run 7), suggesting that the Grignard reagent may function as a chain-shuttling reagent instead hopping between the identical RE SSC catalytic species (Figure 1), while the well-known chain-shuttling reagent ZnEt_2_ for olefin polymerization reactions [36] behaves surprisingly silently (Run 5) in this RE SSC system, and the reason for the latter remains to be clarified.

Under otherwise identical polymerization conditions at ambient temperature and 5 min reaction time, gradually increasing the IP monomer concentration from IP/RE 2000 to 10,000, it is clearly demonstrated that the polymerization reaction can complete in very short times (Runs 3 and 8) when IP concentration is low. When further increasing the IP/RE ratio to 8000 and 10,000, monomer conversion becomes incomplete, and surprisingly, the ultrahigh molecular weight PI (UHMW-PI) appeared at IP/RE ratios of 4000 and above (Run 8, Mw 1036 kg/mol, and Run 9, Mw 2620 kg/mol); when IP/RE increased to 10,000, the MW does not increase as expected, but declines to 916 kg/mol instead, a phenomenon which deviates from living polymerization dynamics and certainly raises another question to study. Another noticeable feature of monomer concentration effects is, at higher IP/RE ratios, the *cis*-1,4-stereoselectivity is gradually increased (96.68% in Run 3, 97.52% in Run 8, 98.84% in Run 9, and over 99% in Run 10) accompanied by gradual vanishing of the *trans*-1,4- and 3,4-units. It is clear that the monomer concentration affects the polymerization reaction in a positive way, i.e., increasing monomer concentration can efficiently increase the molecular weight and *cis*-1,4-selectivity with slightly sacrificing the monomer conversion. The creation of UHMW-PI with the ternary **3**/MMAO-7/Al^i^Bu_3_ catalytic system in appropriate monomer concentration ranges is a meaningful finding for this industrial relevant catalytic system (vide infra).

**Figure 2:**
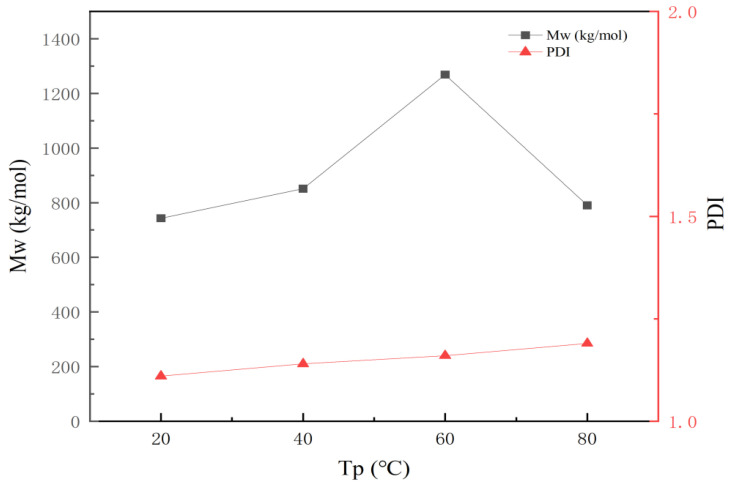
Influences of polymerization temperature on the molecular weight and molecular weight distribution.

**Figure 3:**
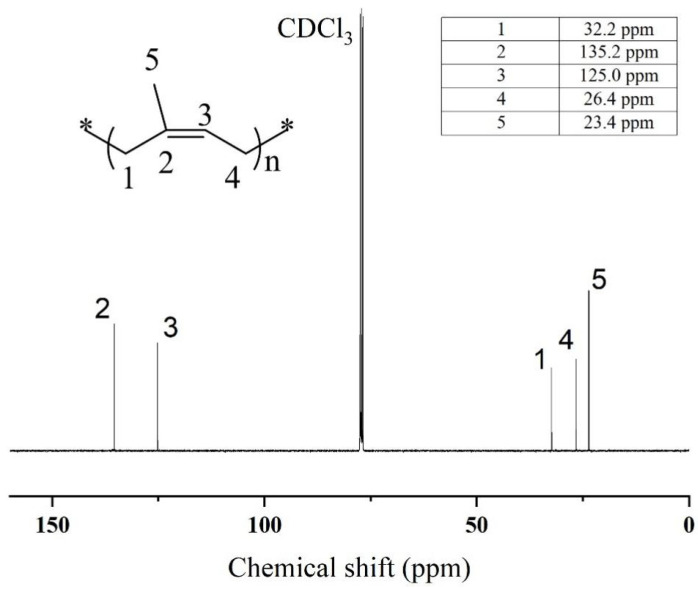
Representative ^13^C NMR spectrum of *cis*-1,4-PI.

In SSC polymerization systems, raising the temperature in a limited range usually facilitates the rates of the polymerization dynamics and the chain-transfer reactions. In the **3**/MMAO-7/Al^i^Bu_3_ catalytic system, under the conditions of an IP/RE ratio of 8000 and 3 min reaction time (keeping incomplete monomer conversion), raising the temperature from ambient to up to 80 °C, the expected increase in monomer conversion is observed (Runs 16–19), suggesting that the catalytic system is thermally stable in a wide temperature range. The system produces very high to ultrahigh molecular weight (743 kg/mol in Run 16 and 1269 kg/mol in Run 18) PIs with very narrow PDIs (1.14–1.19), indicating that living polymerization is in operation in short reaction times in the existence of the IP monomer. It is also interesting to note that when the temperature was raised from ambient to 60 °C, the PI molecular weight also increased with rising temperature (743 kg/mol in Run 16, 851 kg/mol in Run 17 and 1269 kg/mol in Run 18), and with slight broadening of the PDI (from 1.11 in Run 16 to 1.16 in Run 18) (Figure 2). Further raising the reaction temperature to 80 °C resulted in declined molecular weight (790 kg/mol in Run 19 compared with 851 kg/mol in Run 17) and slight broadening of the PDI (1.19 in Run 19 compared with 1.11 in Run 16); again, the *cis*-1,4-selectivities are over 98% to near perfect (>99%). Notice particularly that the *trans*-1,4-unit is not observable, and a representative ^13^C NMR spectrum of *cis*-1,4-PI is shown in Figure 3; the assignment of chemical shifts in the NMR spectrum and the detailed calculation of the microstructure segments are presented in Supporting Information (see Supporting Information S2). It can be roughly concluded that the catalytic system maintained its living nature at temperatures lower than 60 °C, and deviating from the living manner at higher temperatures. In an industrially relevant operation range, temperature has basically a negligible effect on stereoselectivity when the **3**/MMAO-7/Al^i^Bu_3_ catalytic system in operation, a desirable SSC catalytic property for practical application.

At low temperatures and high monomer concentrations, the **3**/MMAO-7/Al^i^Bu_3_ catalytic system is running in typical living polymerization manner (vide supra), and produced *cis*-1,4-PI with a very narrow PDI (1.08–1.21) and gradual increases in molecular weight with increasing reaction time (Runs 20–23), as well the *cis*-1,4-selectivity being near perfection. It is worth noting the shrinking PDI (1.21 in Run 20 and 1.08 in Run 23) with the extension of reaction time (from 30 min in Run 20 to 120 min in Run 23); though the amplitude is not significant, it clearly indicates that the living polymerization feature was maintained for a prolonged reaction period in the existence of an extra IP monomer.

Compared with **1**–**3**/MMAO-3A/Al^i^Bu_3_ catalytic systems [31], the current **1**–**3**/MMAO-7/Al^i^Bu_3_ catalytic system in hexane produces PI macromolecular chains with relatively few *trans*-1,4- and 3,4-units. It is obvious that under the same polymerization conditions, utilizing MMAO-7 co-catalyst is superior to MMAO-3A in stereoselectivity. Moreover, the current **3**/MMAO-7/Al^i^Bu_3_ catalytic system exhibits extremely high catalytic activity with productivity of 6.47 × 10^6^ g/mol·h at ambient temperature (Run 9) and 1.08 × 10^7^ g/mol·h at 80 °C, and provides *cis*-1,4-PI with >98% to perfect stereoselectivity and a very narrow PDI.

## 4. Conclusions

The PNP-MR_2_ complexes (PNP = *o*-dipheylphosphinophenyl)amido, R = *o*-dimethylaminobenzyl, M = Y, **1**; Nd, **2**; Gd, **3**;) were prepared with literature methods. Combining complexes **1**–**3** with commercially available MMAO-7 co-catalyst in saturated hydrocarbons solvent form the homogeneous catalytic systems **1**–**3**/MMAO-7/Al^i^Bu_3_ that exhibit an excellent catalytic property for the coordination polymerization of IP in saturated hydrocarbons. Among these, the **3**/MMAO-7/Al^i^Bu_3_ catalytic system is an outstanding system that showed high productivity up to 1.08 × 10^7^ g/mol·h. The system showed typical living polymerization characteristics in an industrially relevant reaction temperature range. The system delivers high *cis*-1,4-stereoselectivity from 96% to over 99% and very narrow PDIs (1.1–1.6). The system can be operated over an expanded temperature range from 0–80 °C with a negligible temperature effect on the stereoselectivity. The typical chain transfer agent (CTA) ZnEt_2_ does not affect the catalyst performance, while Grignard reagent may function as a chain-shuttling reagent hopping between identical cationic RE SSC metal centers, and HAl^i^Bu_2_ showed regular CTA function in facilitating the catalytic activity and noticeably reducing the *cis*-1,4-PI molecular weight. The **3**/MMAO-7/Al^i^Bu_3_ catalytic system is a new finding with potential practical application in the synthetic rubber industry for the production of high *cis*-1,4-PIs with very high and ultrahigh MW, which could compensate for the shortage of natural rubber supply.

Studies on the polymerization and copolymerization of conjugated dienes and other olefinic monomers by these **1**–**3**/MMAO-7/Al^i^Bu_3_, and related catalyst systems are in progress.

## Figures and Tables

**Table 1:** Polymerization of IP with complexes **1**–**3**/MMAO-7/Al^i^Bu_3_ catalyst systems.

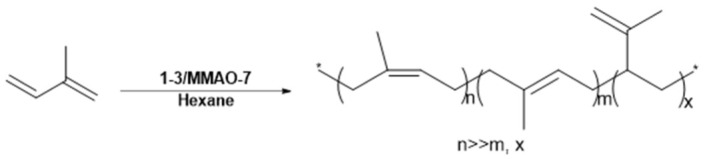											
Run	Cat	IP/RE	P_t_ (min)	T_p_ (°C)	Conversion %	Mw ^a^ (kg/mol)	PDI ^a^	Microstructure ^b^			Tg ^c^ (°C)
								*cis*-1,4%	*trans*-1,4%	3,4-cont.%	
1	1	2000	5	RT	85.1	290	1.42	96.4	/	3.6	−60
2	2	2000	5	RT	62.6	250	1.49	96.1	1.3	2.6	−60
3	3	2000	5	RT	100	378	1.57	96.68	1.35	1.97	−60
4 ^d^	3	2000	5	RT	90.5	644	1.18	99.0	/	1.0	−61
5 ^e^	3	2000	5	RT	30.6	632	1.18	98.6	/	1.4	−60
6 ^f^	3	2000	5	RT	87.4	115	1.74	98.8	/	1.2	−61
7 ^g^	3	2000	5	RT	25.1	687	1.19	98.2	/	1.8	−60
8	3	4000	5	RT	100	1036	1.83	97.52	0.62	1.86	−60
9	3	8000	5	RT	96.3	2620	1.64	98.84	/	1.16	−60
10	3	10,000	5	RT	62.6	916	1.33	>99	/	/	−61
11	3	2000	60	0	100	244	1.30	96.95	1.22	1.83	−60
12	3	2000	30	0	100	266	1.32	97.80	/	2.20	−60
13	3	2000	10	40	100	323	1.23	96.77	1.29	1.94	−60
14	3	2000	3	80	100	335	1.34	96.35	1.46	2.19	−60
15 ^h^	3	2000	10	40	100	211	1.21	98.25	0.58	1.17	−61
16	3	8000	3	RT	62.6	743	1.11	98.62	/	1.38	−61
17	3	8000	3	40	75.5	851	1.14	99.36	/	0.64	−61
18	3	8000	3	60	79.6	1269	1.16	98.20	/	1.80	−60
19	3	8000	3	80	88.5	790	1.19	>99	/	/	−61
20	3	8000	30	0	34.3	964	1.21	>99	/	/	−61
21	3	8000	60	0	70.3	986	1.13	>99	/	/	−61
22	3	8000	90	0	82.1	1061	1.10	>99	/	/	−61
23	3	8000	120	0	88.5	1255	1.08	>99	/	/	−61

Polymerization conditions: IP 1.0 M hexane solution 10 mL (10 mmol), Al^i^Bu_3_/RE = 10, MMAO-7/RE ≥ 200; ^a^ Determined by gel permeation chromatography (GPC) against polystyrene standards; ^b^ Determined by ^1^H NMR spectrum and ^13^C NMR spectrum; ^c^ Measured by differential scanning calorimetry (DSC); ^d n^BuMgEt; ^e^ ZnEt_2_^f^ HAl^i^Bu_2_; ^g^ No chain transfer agent is added; ^h^ polymerization carried out in toluene.

## Data Availability

The original contributions presented in this study are included in the article/Supplementary Materials. Further inquiries can be directed to the corresponding author.

## Supplementary Materials

The following supporting information can be downloaded at: https://www.mdpi.com/article/10.3390/polym17091219/s1, Figure S1: H-1 NMR spectrum of complex 1 (bis(diphenylphosphinyl)amido-Y(-CH2-C6H4-o-NMe2)2; Figure S2: P-31 NMR spectrum of complex 1 (bis(diphenylphosphinyl)amido-Y(-CH2-C6H4-o-NMe2)2).
