# Supplementary material for: Novel High-Efficiency Single-Site Rare Earth (RE) Catalyst System for Isoprene Polymerization
Source: Polymers (Basel). 2025 Apr 29;17(9):1219. doi: 10.3390/polym17091219 (PMC12073132; doi:10.3390/polym17091219)
Supplement: Supplementary file 1 [file polymers-17-01219-s001.zip › polymers-3560530-supplementary.pdf]

polymers

MDPI

Novel High Efficiency Single-Site Rare-Earth （RE） Catalyst System for Isoprene Polymerization

Supporting Information 1

The H-1 and C-13 NMR spectra of complex **1**, (bis(diphenylphosphinyl)amido-Y(-CH_2-C_6H_4-o-NMe_2)$_2$) and the purity of the diamagnetic complex's purity level is sufficiently high for the utilization in subsequent isoprene polymerization experiments.

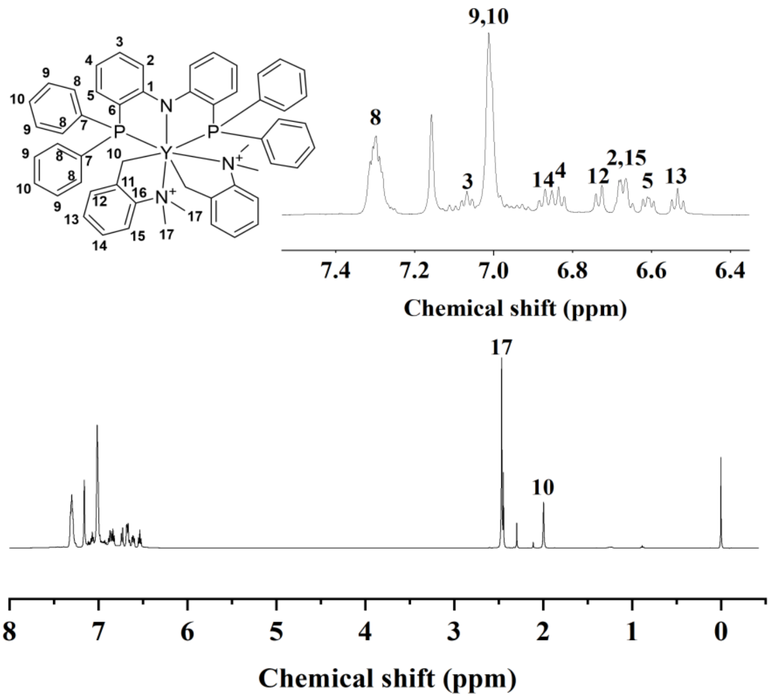

Figure S1. H-1 NMR spectrum of complex **1** (bis(diphenylphosphinyl)amido-Y(-CH_2-C_6H_4-o-NMe_2)$_2$).

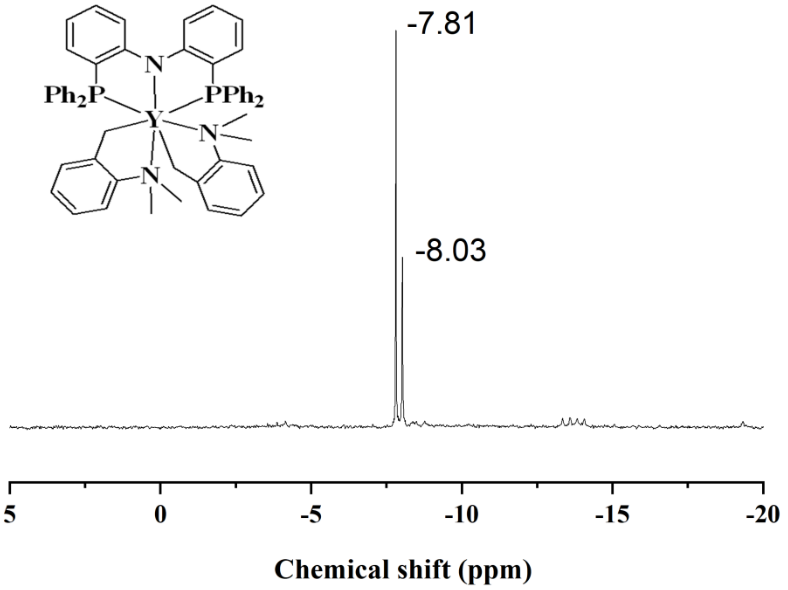

Figure S2. P-31 NMR spectrum of complex **1** (bis(diphenylphosphinyl)amido-Y(-CH_2-C_6H_4-o-NMe_2)$_2$).

For the paramagnetic complexes **2** and **3**, standard synthetic and analytical methods are described in our previous publication. [*Catalysts*, **2022**, *12(10)*, 1131-1145.]

Supporting Information 2

Methods used for cis-1,4-PI, trans-1,4-PI, and 3,4-content, percentage calculation is based on the polymer 13C NMR spectrum signals integration according to the following equations,

Mol% *cis*-1,4-IP% = [**I**23.46/ (**I**23.46 + **I**18.64 + **I**15.98)] × 100% (1)

Mol% *trans*-1,4-IP% = [**I**15.98/ (**I**23.46 + **I**18.64 + **I**15.98)] × 100% (2)

Mol% 3,4-IP% = [**I**18.64/ (**I**23.46 + **I**18.64 + **I**15.98)] × 100% (3)

Where:
I23.46 is the intensity of chemical shift signal at 23.46 PPM
I18.64 is the intensity of chemical shift signal at 18.64 PPM
I15.98 is the intensity of chemical shift signal at 15.98 PPM

For the relevant reference, please see our previous publication in *Catalysts*, **2022**, *12(10)*, 1131-1145.
